# Identification of a chemoresistance-related prognostic gene signature by comprehensive analysis and experimental validation in pancreatic cancer

**DOI:** 10.3389/fonc.2023.1132424

**Published:** 2023-05-12

**Authors:** Junliang Chen, Zhihao Liu, Zhiyuan Wu, Wenjun Li, Xiaodong Tan

**Affiliations:** Department of General Surgery, Shengjing Hospital of China Medical University, Shenyang, Liaoning, China

**Keywords:** pancreatic cancer, chemoresistance, gemcitabine, prognosis, immunotherapy, tumor microenvironment, tumor mutation burden

## Abstract

**Background:**

Chemoresistance is a major hurdle to improving the prognosis of pancreatic cancer (PC). This study aimed to identify key genes regulating chemoresistance and develop a chemoresistance-related gene signature for prognosis prediction.

**Methods:**

A total of 30 PC cell lines were subtyped according to gemcitabine sensitivity data from the Cancer Therapeutics Response Portal (CTRP v2). Differentially expressed genes (DEGs) between gemcitabine-resistant and gemcitabine-sensitive cells were subsequently identified. These upregulated DEGs associated with prognostic values were incorporated to build a LASSO Cox risk model for The Cancer Genome Atlas (TCGA) cohort. Four datasets (GSE28735, GSE62452, GSE85916, and GSE102238) from the Gene Expression Omnibus (GEO) were used as an external validation cohort. Then, a nomogram was developed based on independent prognostic factors. The responses to multiple anti-PC chemotherapeutics were estimated by the “oncoPredict” method. Tumor mutation burden (TMB) was calculated using the “TCGAbiolinks” package. Analysis of the tumor microenvironment (TME) was performed using the “IOBR” package, while the TIDE and “easier” algorithms were employed to estimate immunotherapy efficacy. Finally, RT-qPCR, Western blot and CCK-8 assays were conducted to validate the expression and functions of ALDH3B1 and NCEH1.

**Results:**

A five-gene signature and a predictive nomogram were developed from six prognostic DEGs, including EGFR, MSLN, ERAP2, ALDH3B1, and NCEH1. Bulk and single-cell RNA sequencing analyses indicated that all five genes were highly expressed in tumor samples. This gene signature was not only an independent prognostic factor but also a biomarker forecasting chemoresistance, TMB, and immune cells. *In vitro* experiments suggested that ALDH3B1 and NCEH1 were involved in PC progression and gemcitabine chemoresistance.

**Conclusion:**

This chemoresistance-related gene signature links prognosis with chemoresistance, TMB, and immune features. ALDH3B1 and NCEH1 are two promising targets for treating PC.

## 1 Introduction

Pancreatic cancer (PC) has the highest lethality among all types of malignancies, and its incidence is still growing globally (1). Despite major breakthroughs in the field of oncology, ninety percent of diagnosed patients will eventually succumb to PC within five years (2). With the advancement of multidisciplinary treatment, surgery plus adjuvant chemotherapy is increasingly important in cancer management. In addition, neoadjuvant chemotherapy can potentially eliminate undetectable micrometastases and decrease the margin positivity rates of resectable and borderline resectable PC patients (3). Insidious onset and rapid progression are substantial hurdles limiting the opportunity for radical resection and survival outcomes of PC patients. Chemotherapy-based regimens are one of the most recommended strategies for tumor downstaging and conversion to resectability (4). Thus, how to increase chemosensitivity is closely related to improving the prognosis of PC patients.

Gemcitabine, chemically known as 2′,2′-difluorodeoxycytidine, is a DNA synthesis inhibitor that has been widely used as a first-line anti-PC drug since 1997 (5). However, its overall survival (OS) benefit is less than satisfactory. Hypovascularized stroma accounts for approximately 90% of the total tumor volume and serves as an important factor that prevents gemcitabine delivery into the cancerous lesion (6). Targeting the tumor microenvironment (TME) is an appealing approach to increase the gemcitabine response rate. For example, nanoparticle albumin-bound paclitaxel (nab-paclitaxel) synergizes with gemcitabine to improve the OS of PC patients by depleting the peritumoral stroma (7). Although gemcitabine is superior to other antineoplastic drugs as monotherapy in PC, rapid development of resistance is another urgent issue that remains to be solved (8). It is therefore highly desirable to discover new targets to increase options for combination therapy and promote susceptibility to gemcitabine.

In the present study, we identified six prognostic genes (ALDH3B1, CHST11, EGFR, ERAP2, MSLN, and NCEH1) that were upregulated in gemcitabine-resistant PC cells compared with gemcitabine-sensitive PC cells. Then, we explored the expression levels of the six genes in bulk RNA sequencing (RNA-seq) and single-cell RNA sequencing (scRNA-seq). A novel prognostic gene signature and nomogram were developed based on The Cancer Genome Atlas (TCGA) cohort and validated externally in the Gene Expression Omnibus (GEO) cohort. Further analyses revealed that this signature was related not only to gemcitabine chemoresistance but also to therapy responses to other first-line anti-PC drugs, tumor mutation burden (TMB), and immune checkpoint blockades (ICBs). The results of CCK-8 assays indicated that ALDH3B1 and NCEH1 were new therapeutic targets for enhancing gemcitabine sensitivity and restraining cancer growth. Overall, this chemoresistance-related signature opens new avenues for prognosis assessment and personalized medication for PC patients.

## 2 Materials and methods

### 2.1 Data acquisition and processing

The raw drug response data of the Cancer Therapeutics Response Portal (CTRP v2) were obtained from the CTD² Data Portal (https://ocg.cancer.gov/programs/ctd2/data-portal/). The RNA-seq data of corresponding pancreatic cancer cell lines are available from the DepMap portal (https://depmap.org/portal/). The “TCGAbiolinks” R package (9) was used to download and process the bulk RNA-seq results of 178 PC patients along with clinical, mutation, and copy number variation (CNV) files in the TCGA database. The TCGA cohort was used as the training cohort to develop a predictive gene signature and nomogram. Meanwhile, a total of 186 patients with complete follow-up information from the GEO (https://www.ncbi.nlm.nih.gov/geo/) database were merged as the validation cohort, including GSE28735, GSE62452, GSE85916 and GSE102238 datasets.

As there are no data regarding normal pancreatic cell lines in the DepMap portal, the transcriptome data of normal pancreatic duct epithelial cell lines (HPNE and HPDE) were obtained from GSE97003 and GSE181625. We also explored the expression levels of key genes regulating gemcitabine resistance between normal and tumor samples at both bulk and single-cell resolutions. Two scRNA-seq datasets were included in our study, GSE212996 and CRA00160 (10). The sequencing data of normal pancreatic tissues from the GTEx portal (https://gtexportal.org/home/) were analyzed in combination with the TCGA cohort. We also compared the expression levels of these genes in matched tumor-normal samples from GSE28735 (45 pairs), GSE102238 (28 pairs), and GSE101448 (18 pairs).

Raw RNA-seq and microarray data of bulk tissues were normalized using transcripts per kilobase million (TPM) transformation and robust multichip average (RMA) normalization, respectively, followed by log2 (x + 1) conversion (11, 12). The “Seraut” package was used to create an S4 object from the unique molecular identifier (UMI) count and barcode matrices of scRNA sequencing in accordance with the standard pipeline (13). After excluding low-quality cells (< 200 genes/cell, > 3000 genes/cell, < 3 cells/gene, hemoglobin genes < 3%, and > 10% mitochondrial genes), linear principal component analysis (PCA) and nonlinear uniform manifold approximation and projection (UMAP) algorithm were adopted to visualize the clustering results. The primary cell type annotations of the “singleR” package (14) were checked and adjusted manually based on marker genes from the CellMarker database (http://xteam.xbio.top/CellMarker/).

### 2.2 Identification of key genes regulating gemcitabine sensitivity

A lower gemcitabine resistance score indicates a higher drug sensitivity. According to the scaled gemcitabine resistance score, pancreatic cells were subtyped into gemcitabine-sensitive (Z score ≤ –0.5), intermediate gemcitabine-resistant (–0.5< Z score < 0.5), and gemcitabine-resistant (0.5 ≤ Z score) groups. Subsequently, the “limma” package was used to identify differentially expressed genes (DEGs) between gemcitabine-sensitive and gemcitabine-resistant cells (15). The DEGs with a *P* value < 0.01 and log_2_ fold change (FC) > 1 were defined as the key regulators contributing to gemcitabine resistance.

### 2.3 Risk model development and evaluation

The least absolute shrinkage and selection operator (LASSO) Cox regression algorithm was applied to develop a risk model from the prognostic genes associated with chemoresistance in the TCGA cohort (16). The optimal penalization parameter was selected by tenfold cross validation. Gene signature with better performance and fewer included genes was selected as the appropriated model from multiple runs. The risk score of each enrolled patient was equal to the sum of each gene expression value multiplied by the corresponding coefficient. In line with other studies, the median risk score was selected as the threshold to equally divide the patients in the training cohort into two groups (17). Utilizing the “survival”, “survminer”, “timeROC”, and “stats” R packages, the prognostic values in the TCGA, GEO, and whole cohort were evaluated by Kaplan-Meier (K-M) curves with log-rank tests, time-dependent receiver operating characteristic (tROC) curves, and principal component analyses (PCA).

### 2.4 Nomogram construction and validation

Univariate and multivariate analyses were conducted to identify independent prognostic factors with statistical significance for constructing a predictive nomogram (18). The performance of the nomogram was assessed by the concordance index (C index), tROC, decision curve analysis (DCA), and calibration curve. The following R packages were used for analyses and visualization: “survival”, “rms”, “pec”, and “ggDCA”.

### 2.5 Functional enrichment analysis and TMB calculation

Comparison of functional enrichment scores between the high- and low-risk groups was implemented by the “GSVA” and “limma” packages. Gene sets for GSVA analyses are open-access from the Molecular Signatures Database (MSigDB; https://www.gsea-msigdb.org/gse), including Hallmark (c2.all.v2022.1.Hs.symbols) and KEGG (c2.KEGG.v2022.1.Hs.symbols) collections. TMB in the TCGA cohort was calculated as the number of mutated bases per million bases utilizing the “TCGAbiolinks” package.

### 2.6 Chemotherapy response prediction

The “calcPhenotype” function of “oncoPredict” R package was used to predict the drug sensitivity for each patient based on bulk RNA-seq result (19). The datasets for fitting the ridge regression model are updated and uploaded by the “oncoPredict” team, including resources from the Genomics of Drug Sensitivity in Cancer (GDSC2) and CTRP V2 databases (https://osf.io/c6tfx/).

### 2.7 TME deconvolution and immunotherapy response estimation

The deconvolution module of the “IOBR” R package (20) was used to estimate the landscape of the TME, which integrates six open-source deconvolution methodologies, namely, CIBERSORT (21), MCPcounter (22), EPIC (23), xCell (24), quantiseq (25), and TIMER (26). In addition, the ssGSEA algorithm was also adopted to evaluate the TME score for each patient (27). The reference gene sets for ssGSEA estimation were gathered from previous works (28–32).

First, the ICB resistance score was calculated using the Tumor Immune Dysfunction and Exclusion (TIDE) algorithm (32). However, the TIDE signature may be biased, as only tumor-infiltrating cell signatures are incorporated for estimation. Generally, the immune response (immune cells, intracellular networks, and intercellular networks) and tumor antigenicity (TMB) are two hallmarks involved in regulating the response to immunotherapy (33). Then, the Estimate Systems Immune Response (EaSIeR) method was introduced to predict ICB response, taking into account the tumor microenvironment as a whole (34). A lower TIDE value and a higher EaSIeR score indicate a better ICB response.

### 2.8 Cell culture and transient transfection

Three human pancreatic cancer cell lines (AsPC-1, CFPAC-1, and PANC-1) were purchased from Procell Life Science & Technology Co., Ltd. (Wuhan, China). Human pancreatic normal cell lines (hTERT-HPNE) were obtained from Meisen Cell Biotechnology Co., Ltd. (Hangzhou, China). AsPC-1 and CFPAC-1 cells were maintained in Roswell Park Memorial Institute-1640 and Iscove’s Modified Dulbecco’s Medium (IMDM; Procell), respectively. PANC-1 and HPNE cells were cultivated in high-glucose Dulbecco’s modified Eagle’s medium (DMEM; Procell). All media contained 10% fetal bovine serum (FBS; Procell) and 100 μg/mL penicillin-streptomycin solution (Procell). The humidity incubator was set to 37°C with 5% CO2.

Cells in six-well plates were transiently transfected with siRNA using Lipofectamine™ 3000 transfection reagent (Invitrogen, Carlsbad, CA, USA) according to the manufacturer’s protocol. siRNAs were synthesized by GenePharma (Shanghai, China), including ALDH3B1 siRNA (sequence: 5′-GCU GAA GCC AUC GGA GAU UAG tt-3′), NCEH1 siRNA (sequence: 5′-CAA UGA UCG UUA ACA AUC Att-3′), and universal negative control (NC) siRNA.

### 2.9 RNA isolation and quantitative real-time PCR

Total RNA was isolated using RNA Isolater Total RNA Extraction Reagent (Vazyme, Nanjing, China) and dissolved in RNase-free ddH_2_O (Vazyme). Reverse transcription and quantitative real-time PCR (qRT-PCR) procedures were performed using Hiscript III Reverse Transcriptase (Vazyme) and ChamQ Universal SYBR qPCR Master Mix (Vazyme). The primers were synthesized by Sangon (Shanghai, China), and the sequences were as follows: ALDH3B1, 5′-GCC CTG GAA CTA TCC GCT G-3′ (forward), 5′-CGT TCT TGC TAA TCT CCG ATG G-3′ (reverse); NCEH1, 5′-GAA TAC AGG CTA GTT CCA AAG-3′ (forward), 5′-TAC TTC TGT AAG ACT TCT GGC-3′ (reverse); GAPDH, 5′-GGA GCG AGA TCC CTC CAA AAT-3′ (forward), 5′-GGC TGT TGT CAT ACT TCT CAT GG-3′ (reverse). The whole experimental process was carried out as described previously by our group (35). Gene expression was compared using the semiquantitative 2-^ΔΔCt^ method.

### 2.10 Western blot analysis

Western blotting (WB) was performed according to the previous protocol (36). In brief, total protein was extracted using RIPA buffer (Sevenbio, Beijing, China), denatured for 10 min in a dry bath, and then quantified using BCA kit (Sevenbio).A total of 20ug protein was loaded for 10% SDS-PAGE electrophoresis and transferred to PVDF membrane (Millipore, Hertfordshire, UK), which was then blocked by 5% non-fat milk. After overnight incubation with primary antibodies at 4°C, the PVDF membrane was incubated with secondary antibodies for 2 h at room temperature. Signals were determined using ECL kit (Sevenbio). Primary antibodies against ALDH3B1 (1:500, Zen Bioscience, Chengdu, China), NCEH1 (1:500, Zen Bioscience), and β-actin (1:10000, ProteinTech Group, Rosemont, IL, USA) were used. Gray value analysis of all bands was performed using Image J software (Image J 1.53, NIH).

### 2.11 Cell proliferation and cytotoxicity assays

Cell Counting Kit-8 (CCK-8; GlpBio, CA, USA) was used to measure cell viability and proliferation. The experimental procedure was the same as the official instructions. For the proliferation assay, transfected cells (2000 cells per well) were seeded in 96-well plates, and optical density (OD) 450 nm values were measured at 0, 24, 48, 72, 96, and 120 hours with a microplate reader (BioTek, Vermont, USA). Cell proliferation fold was calculated using the following method: cell proliferation fold = (OD_0-120 hours_ – OD_blank_)/(OD_0 hours_ – OD_blank_). For the cytotoxicity assay, 4000 cells per well were plated in 96-well plates 24 hours before gemcitabine (GlpBio) treatment. After 48 hours, OD values were determined, and cell viability was obtained using the following algorithm: cell viability = (OD_gemcitabine-treated group_ – OD_blank_)/(OD_control group_ – OD_blank_).

### 2.12 Statistical analysis

All statistical analyses and graphical visualizations were implemented using R software (version 4.21) and packages from the Comprehensive R Archive Network (CRAN) or Bioconductor repositories. Two-tailed Student’s t test, nonparametric Wilcoxon, and Spearman tests were used for comparison and correlation analyses, as appropriate. Two-tailed *P* < 0.05 was considered significant.

## 3 Results

### 3.1 DEGs upregulated in gemcitabine-resistant PC cell lines

**Figure 1:**
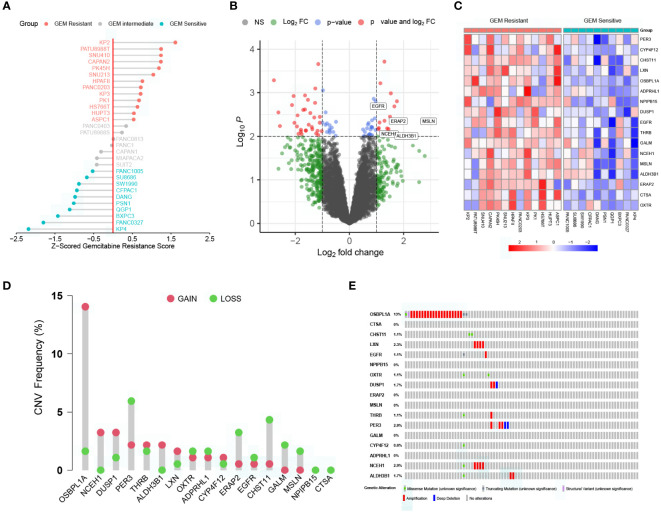
Characterization of key genes regulating gemcitabine resistance. **(A)** Scaled gemcitabine resistance scores of the 30 pancreatic cancer cell lines in the CTRP database. **(B)** Differentially expressed genes (DEGs) between gemcitabine-sensitive and gemcitabine-resistant cell lines. **(C)** Heatmap depicting the expression levels of the 17 DEGs correlated with gemcitabine resistance. **(D)** Copy number variation (CNV) frequencies of the 17 DEGs in the TCGA cohort. **(E)** Somatic mutation frequencies of the 17 DEGs in the TCGA cohort.

The workflow of the current study is presented in **Supplementary Figure S1**. To better compare the differences in gemcitabine sensitivity, data from the CTRP database were first Z-scored. As similar responses were observed in seven cell lines (PANC0403, PATU8988S, PANC0813, PANC1, CAPAN1, MIAPACA2, and SUIT2), 0.5 and –0.5 were selected as the grouping thresholds. Of the 30 PC cell lines, 13 were classified as gemcitabine-resistant, 7 as intermediate gemcitabine-resistant, and 10 as gemcitabine-sensitive (**Figure 1A** and **Supplementary Figure S2**). Differential expression analysis revealed that the expression levels of 17 genes were significantly higher in the gemcitabine-resistant group than in the gemcitabine-sensitive group (**Figures 1B, C**). Additionally, we summarized the CNV and somatic mutation frequencies in the TCGA cohort (**Figures 1D, E**). The number of CNV gains was much higher than CNV losses in OSBPL1A, NCEH1, DUSP1, and ALDH3B1, while the opposite tendencies were observed in ERAP2, CHST11, GALM, and MSLN. The mutation rates of the DEGs related to gemcitabine chemoresistance were less than 3%, except for OSBPL1A (13%).

### 3.2 Expression levels of the prognostic genes related to gemcitabine chemoresistance measured by bulk RNA-seq and scRNA-seq

**Figure 2:**
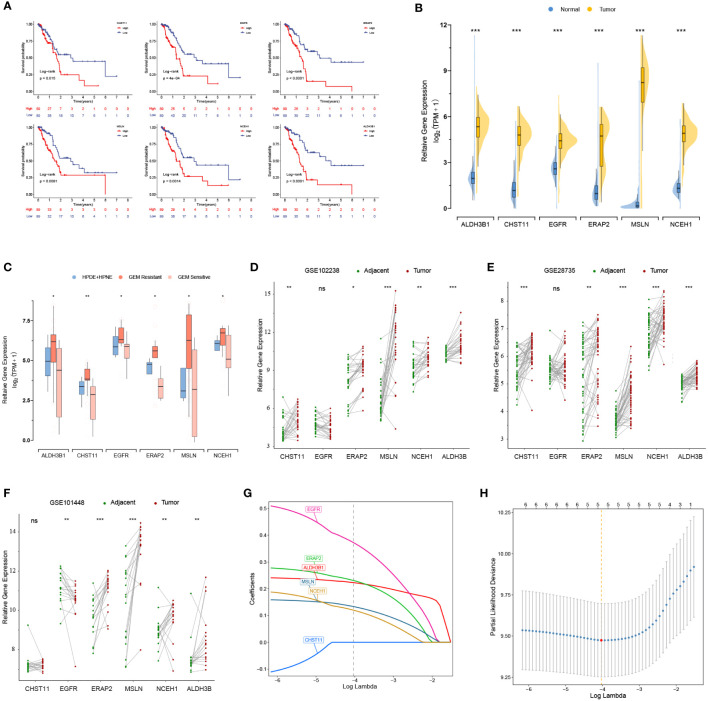
Prognostic values and expression levels of the genes related to gemcitabine resistance. **(A)** K-M analyses of the six differentially expressed genes (DEGs) associated with gemcitabine chemoresistance and prognosis in the TCGA cohort. **(B-F)** Comparison of the expression levels of the six prognostic DEGs in the GTEx-TCGA cohort **(B)**, pancreatic cell lines **(C)**, and matched tumor-normal samples **(D-F)**. **(G, H)** LASSO Cox analysis. The optimal number of signatures was determined by tenfold cross-validation. *p < 0.05; **p < 0.01; ***p < 0.001; ns, not statistically significant.

K-M analysis revealed that 6 out of 17 DEGs associated with gemcitabine chemoresistance had prognostic value in the TCGA cohort, namely, ALDH3B1, CHST11, EGFR, ERAP2, MSLN, and NCEH1 (**Figure 2A**). The expression levels of the six prognostic DEGs were significantly higher in the TCGA tumor samples than in the nonmatched GTEx normal samples (**Figure 2B**). Consistently, these genes were more highly expressed in gemcitabine-resistant PC cell lines than in normal pancreatic cell lines (**Figure 2C**). However, matched tumor-normal comparisons indicated that EGFR was not relatively upregulated in malignant tissues (**Figures 2D-F**).

**Figure 3:**
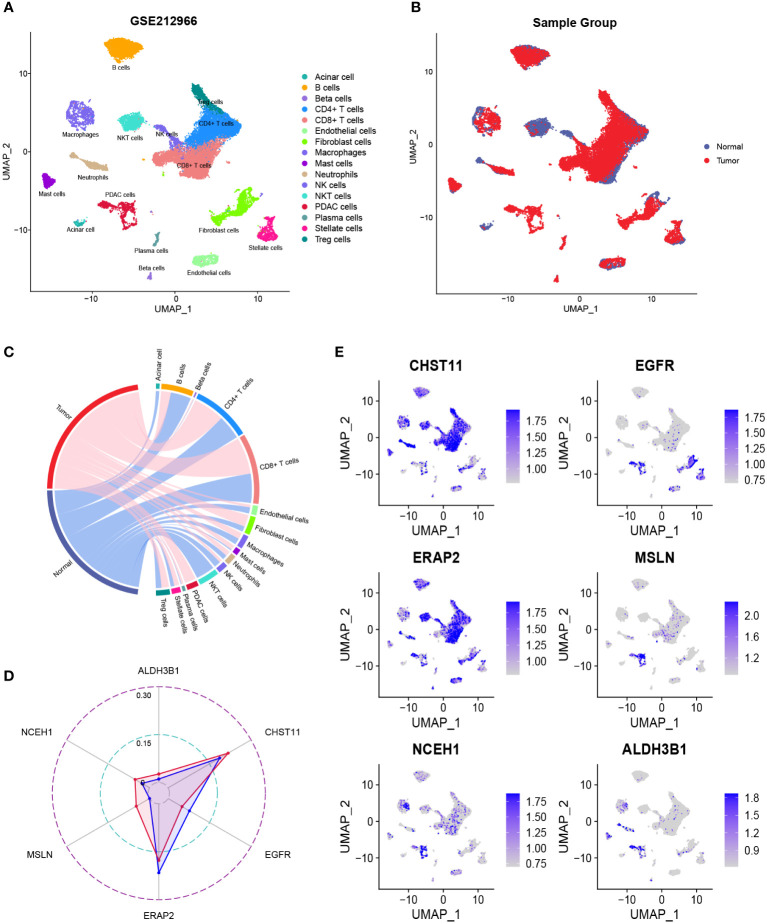
The dimension reduction of the GSE212966 dataset. **(A, B)** Uniform manifold approximation and projection (UMAP) plots of single-cell RNA sequencing. **(C)** Percentage of the detected cells in tumor and normal samples. **(D)** Comparison of the mean unique molecular identifier (UMI) counts between tumor and normal samples. **(E)** Feature plots of CHST11, EGFR, ERAP2, MSLN, NCEH1, and ALDH3B1.

**Figure 4:**
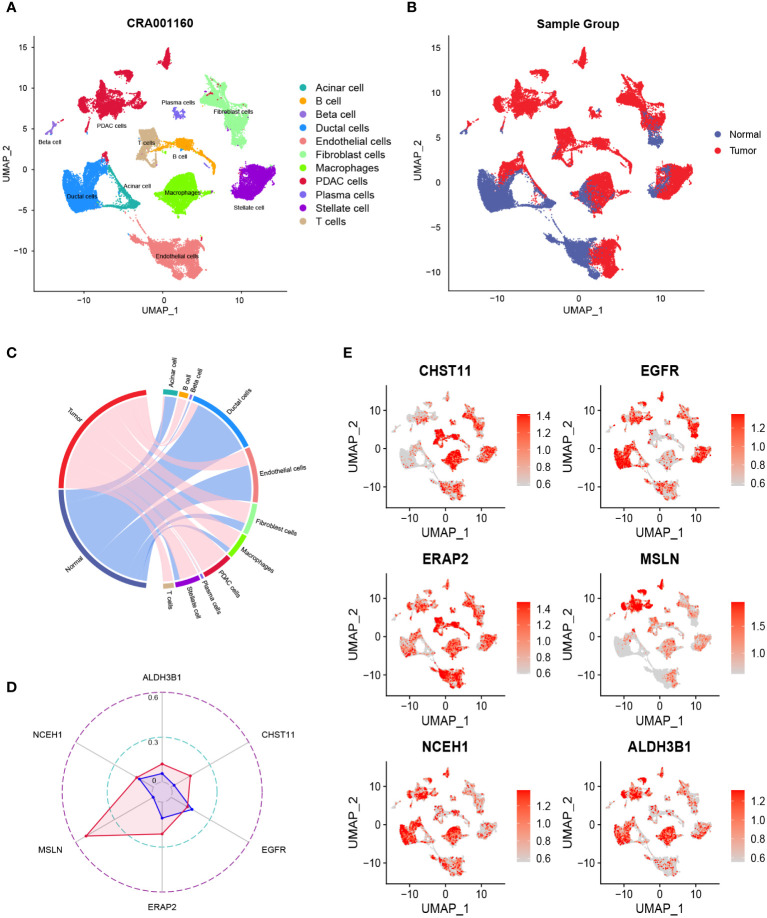
The dimension reduction of the CRA00160 dataset. **(A, B)** Uniform manifold approximation and projection (UMAP) plots of single-cell RNA sequencing. **(C)** Percentage of the detected cells in tumor and normal samples. **(D)** Comparison of the mean unique molecular identifier (UMI) counts between tumor and normal samples. **(E)** Feature plots of CHST11, EGFR, ERAP2, MSLN, NCEH1, and ALDH3B1.

After quality control, the number of normal/tumor cells in GSE212966 was 10751/17338 and in CRA00160 was 13771/34974. At the single-cell level, tumor cells expressed low levels of CHST11 compared with ALDH3B1, EGFR, ERAP2, MSLN, and NCEH1 (**Figures 3A-E**, **4A-E**). The fibroinflammatory stroma and cancer-associated fibroblast (CAF)-enriched TME are two characteristics of PC (37). Pancreatic stellate cells are the precursors of CAFs, which are essential in tumorigenesis and gemcitabine resistance (37, 38). As shown in the UMAP plots, most of the stellate and fibroblast cells came from the tumor samples (**Figures 3A-C**, **4A-C**). The relatively low expression of EGFR in bulk RNA-seq analyses was further confirmed by single-cell analyses (**Figures 3D**, **4D**). However, it was still upregulated in malignant and stromal components of tumor tissues, proving that it was indispensable for tumor progression (**Figures 3E**, **4E**). In contrast to the results in CRA00160, the mean UMI count of ERAP2 in GSE212966 was lower in tumor samples, which could be accounted for by the low proportion of detected malignant cells (**Figures 3A-D**).

### 3.3 Development and validation of a chemoresistance-related gene signature

**Figure 5:**
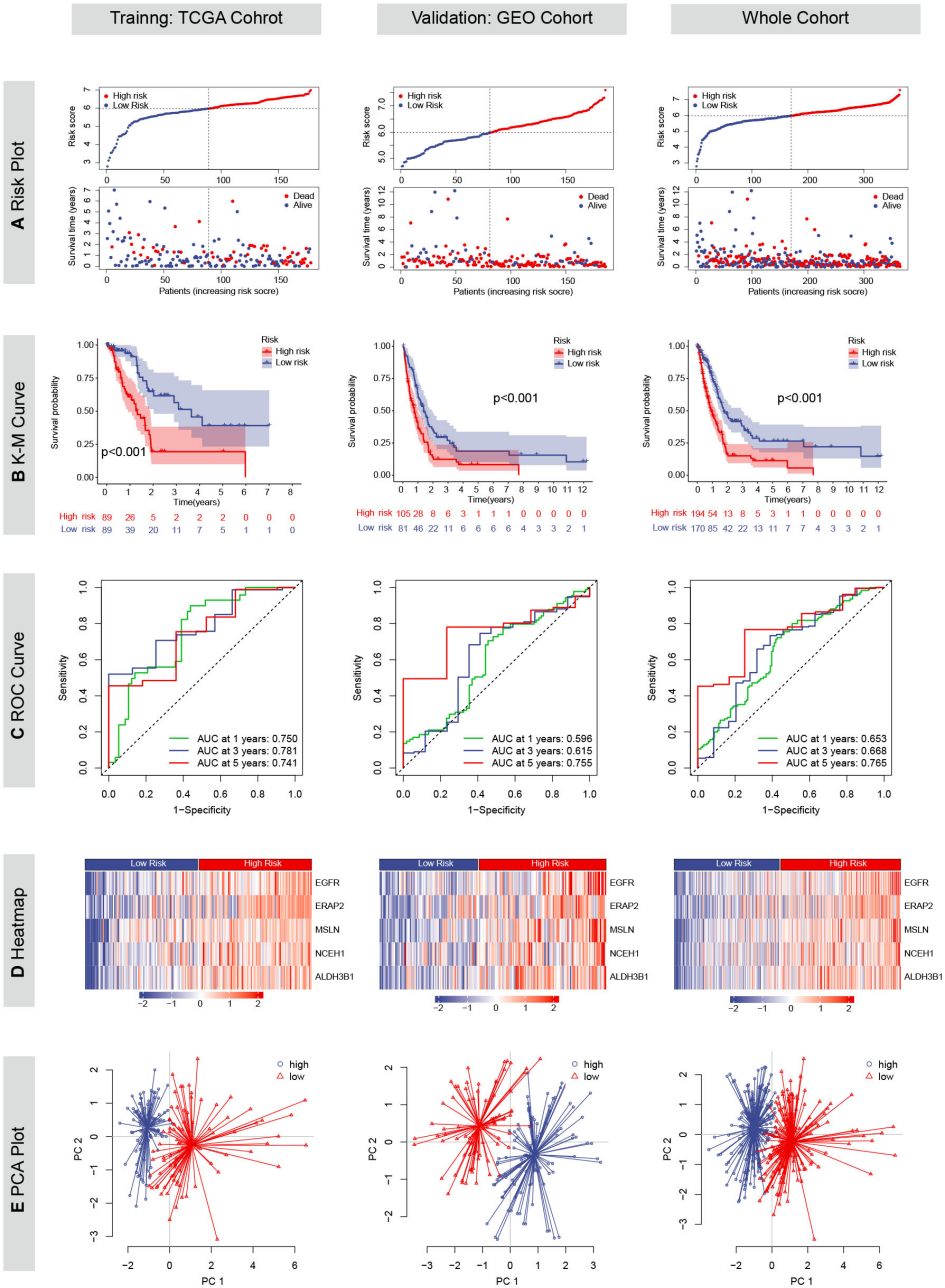
Prognostic values of the chemoresistance-related signature in TCGA, GEO and whole cohorts. **(A)** Risk plots depicting the risk score and survival status distributions. **(B)** Kaplan-Meier (KM) curves comparing overall survival between high- and low-risk groups. The survival difference was evaluated by the log-rank test. **(C)** Time-dependent receiver operating characteristic (ROC) curves and area under the curve (AUC) analyses for predicting 1-, 3-, and 5*-*year survival. **(D)** Heatmaps showing the Z-scored expression of the five genes constituting the risk model. **(E)** Principal component analyses (PCA).

Except for CHST11, the other five chemoresistance-related genes were included in the LASSO Cox model predicting the OS of the TCGA training cohort (**Figures 2G, H**). The risk score formula was as follows: risk score = (0.3731 * EGFR expression) + (0.2364 * ERAP2 expression) + (0.1588 * MSLN expression) + (0.1254 * NCEH1 expression) + (0.2233 * ALDH3B1 expression). On the basis of the median risk score of the patients in the TCGA cohort, all the enrolled patients were separated into high- or low-risk groups. K-M analyses and risk plots indicated that patients with higher risk scores had significantly worse prognoses (**Figures 5A, B**). Log-rank test *P* values of the TCGA, GEO, and whole cohorts were less than 0.001. The prediction accuracies of the 1-, 3-, and 5-year survival rates were 0.750/0.781/0.741 in the TCGA cohort, 0.596/0.615/0.755 in the GEO cohort, and 0.653/0.668/0.765 in the whole cohort, respectively (**Figure 5C**). Positive correlations were observed between the risk score and expression levels of the five genes (**Figure 5D**). Patients in different risk groups could be clearly distinguished in PCA reduction diagrams (**Figure 5E**).

### 3.4 Establishment of a predictive nomogram based on key genes regulating gemcitabine chemoresistance

**Figure 6:**
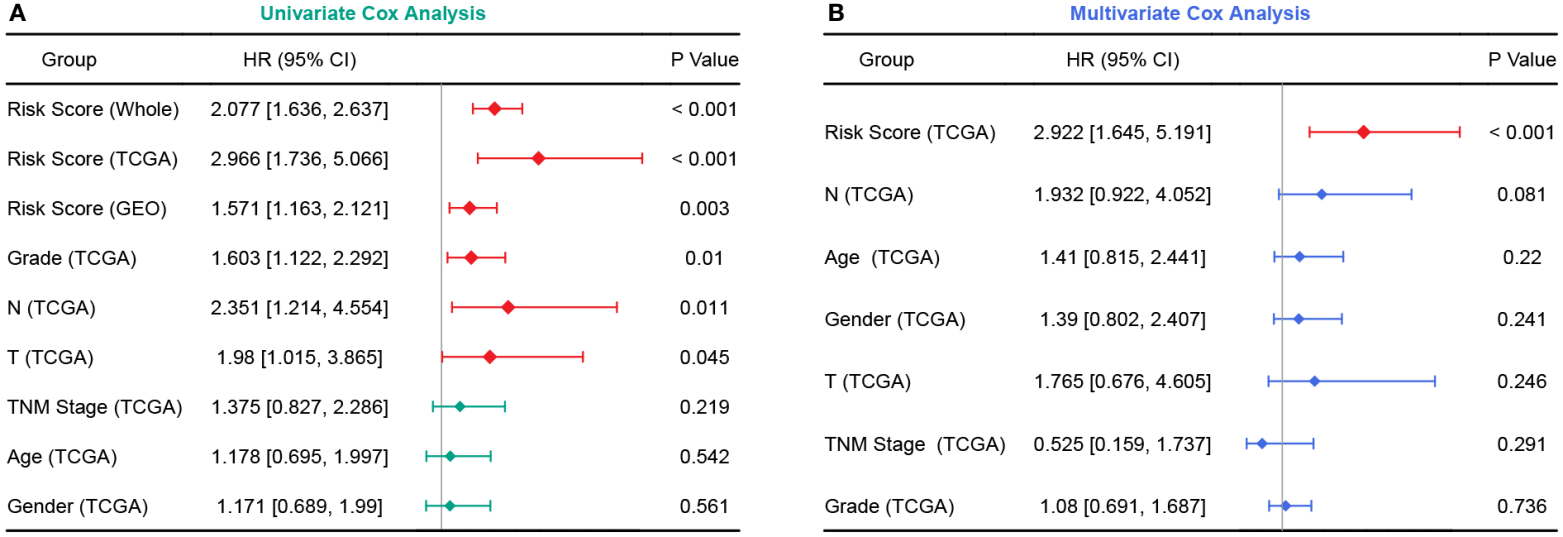
Forest plots of univariate and multivariate Cox analyses. **(A)** Univariate Cox analyses in the TCGA, GEO, and whole cohort. **(B)** Multivariate Cox analysis in the TCGA cohort.

**Figure 7:**
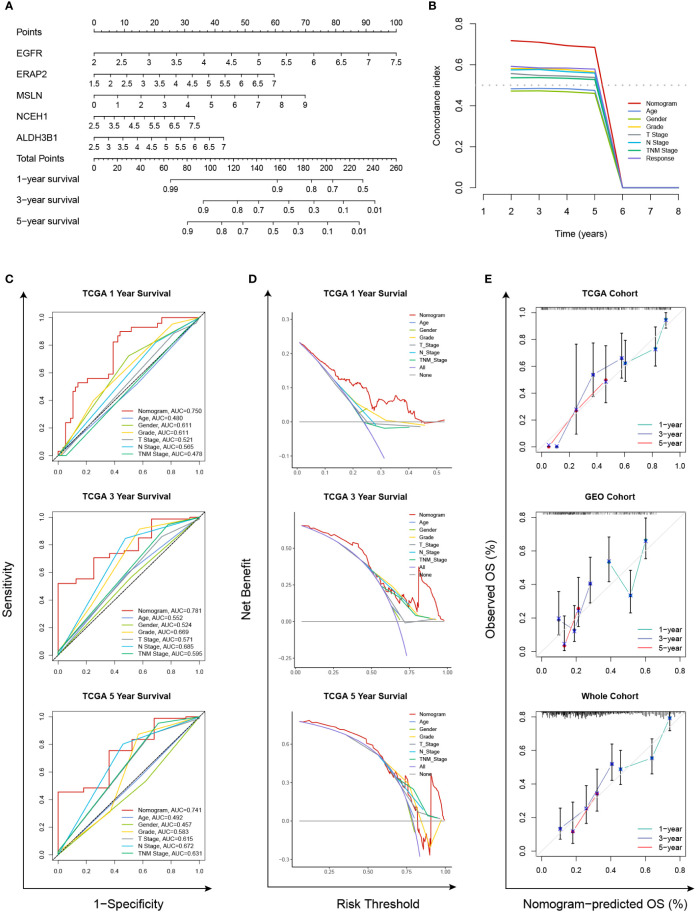
Establishment and validation of a chemoresistance-related nomogram. **(A)** A predictive nomogram based on the expression levels of EGFR, ERAP2, MSLN, NCEH1, and ALDH3B1. **(B)** Concordance index analyses of clinicopathological factors in the TCGA cohort. **(C–E)** Evaluations of the prediction performance by concordance index (C index), time-dependent receiver operating characteristic (tROC), and decision curve analysis (DCA).

In the TCGA cohort, the chemoresistance-related risk score was the only independent prognostic factor identified by univariate and multivariate Cox analyses (**Figures 6A, B**). In addition, the risk score was also a significant risk factor in the GEO and whole cohorts (**Figure 6A**). As the clinicopathological information of the GEO patients was incomplete, multivariate Cox regression was not applicable. Therefore, a nomogram predicting 1-, 3-, and 5-year OS rates was built based on the expression levels of the five genes constituting the risk model (**Figure 7A**). C index analysis indicated that the nomogram had the highest prediction performance compared with other factors in the TCGA cohort, which was further supported by tROC and DCA curves (**Figures 7B-D**). Then, the accuracies of the 1-, 3-, and 5-year OS predictions were validated internally and externally, and the deviations between the nomogram-predicted and observed OS were small (**Figure 7E**).

### 3.5 Chemosensitivity analyses

**Figure 8:**
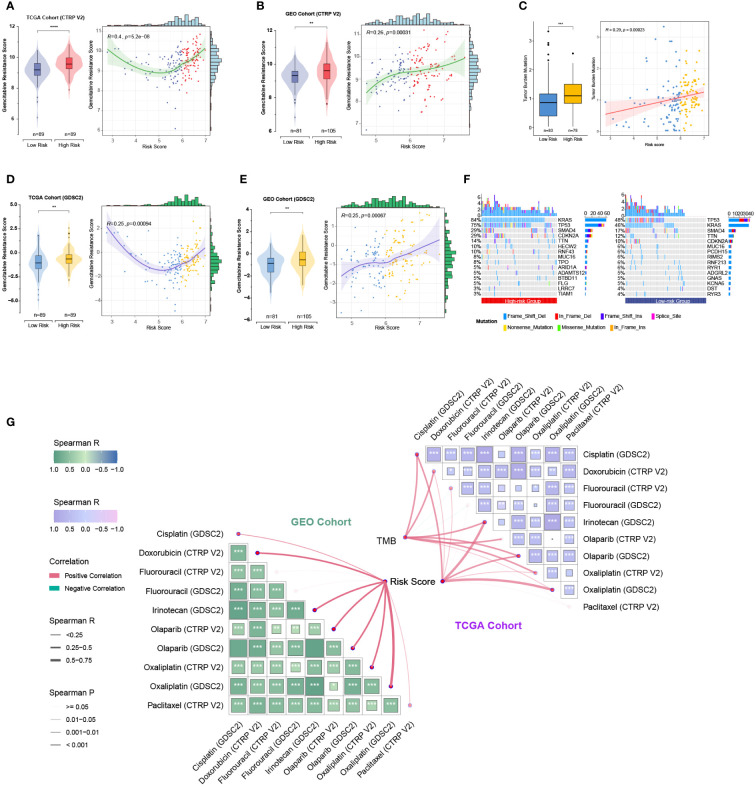
Relationships among the chemoresistance-related risk score, tumor mutation burden, and estimated chemotherapeutic responses to anti-pancreatic cancer agents. **(A-D)** Gemcitabine resistance scores of TCGA and GEO cohorts were estimated by the “oncoPredict” R package using CTRP V2 **(A, B)** or GDSC2 **(C, D)** data as the training cohort. **(E)** Correlation of the five-gene signature with tumor mutation burden (TMB) level in the TCGA cohort. **(F)** Oncoplots showing the top 15 most altered genes between the high- and low-risk groups of the TCGA cohort. **(G)** Correlation matrices depicting the relationships among the chemoresistance-related risk score, TMB, and therapy responses to first-line chemotherapeutics. **, p < 0.01; ***, p < 0.001; ****, p < 0.0001.

As the five prognostic genes (ALDH3B1, EGFR, ERAP2, MSLN, and NCEH1) were highly expressed in gemcitabine-resistant PC cell lines, this established gene signature was, therefore, a very promising indicator of gemcitabine sensitivity for PC patients. This hypothesis was supported by subsequent analyses, which showed that the risk score increased with the gemcitabine resistance score, and the low-risk group was more sensitive to gemcitabine (**Figures 8A-D**). TMB is a genetic characteristic of cancers. In the TCGA cohort, the risk score was positively correlated with TMB (**Figure 8E**). The top five mutated genes in the high- and low-risk groups were KRAS (84%/46%), TP53 (75%/48%), SMAD4 (29%/17%), CDKN2A (29%/12%), and TTN (14%/10%) (**Figure 8F**). Since this gene signature was closely related to gemcitabine sensitivity, we further explored its potential in predicting the response to other commonly used anti-PC drugs. As shown in **Figure 8G**, patients with higher risk scores were more likely to be resistant to doxorubicin, olaparib, paclitaxel, and FOLFRINOX components (fluorouracil, irinotecan, oxaliplatin), while the same tendencies were also observed in patients with higher TMB levels.

**Figure 9:**
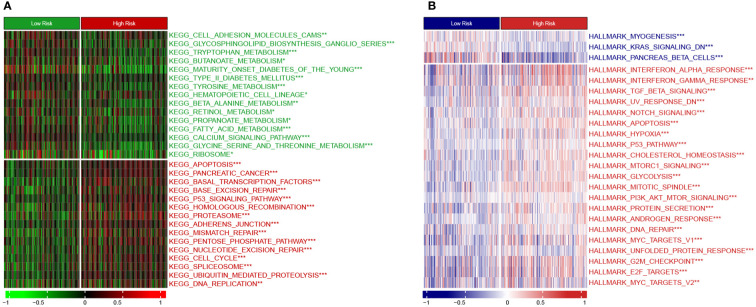
Heatmap showing GSVA enrichment scores **(A)** KEGG pathway analysis. **(B)** Hallmark analysis. *, p < 0.05; **, p < 0.01; ***, p < 0.001.

Moreover, we employed the GSVA method to understand the mechanisms underlying chemoresistance. The pathways enriched in the high-risk group were mainly associated with pancreatic cancer, apoptosis, mismatch repair, cell cycle, spliceosome, hypoxia, glycolysis, P53 signaling, MYC signaling, TGF-beta signaling, and PI3K/Akt signaling (**Figures 9A, B**). The low-risk group showed enrichment for pathways related to metabolism and downregulation of KRAS. Thus, hyperactivation of oncogenic pathways could account for the dismal prognosis and chemoresistance in the high-risk group. Taken together, this gene signature was a versatile tool favoring multidrug resistance prediction.

### 3.6 Evaluations of immune infiltration and immunotherapy sensitivity

**Figure 10:**
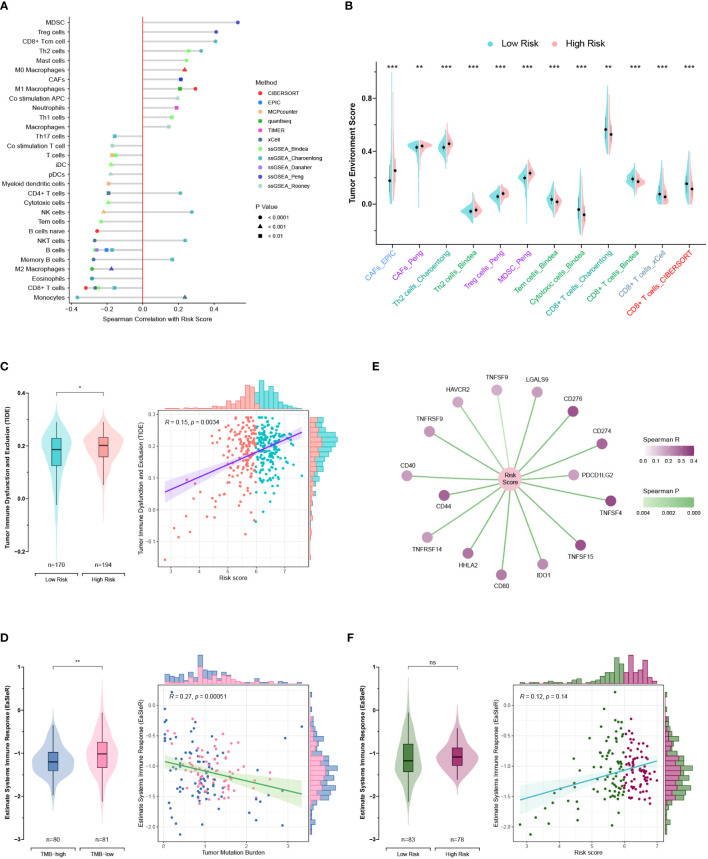
Exploration of the tumor microenvironment and estimation of immunotherapy efficacy. **(A)** Estimation of tumor-infiltrating cells for TCGA and GEO patients. This analysis was performed using the CIBERSORT, MCPcounter, EPIC, xCell, quantiseq, TIMER, and ssGSEA algorithms. **(B)** Comparison of the tumor environment score between the high- and low-risk groups. **(C)** Assessment of therapy response to immune checkpoint blockade (ICB) using the TIDE algorithm for TCGA and GEO patients. **(D)** Correlations of the risk score with immune checkpoints. **(E, F)** Correlations of the EaSIeR score with the tumor mutation burden (TMB) **(E)** and risk score **(F)**. Only TCGA patients with available TMB data were included in the EaSIeR score calculation. ns, not significant; *, p < 0.05; **, p < 0.01; ***, p < 0.001.

Seven published methods were integrated to comprehensively evaluate the TME compositions of PC patients in TCGA and GEO cohorts. The risk score exhibits positive correlations with cells favoring immunosuppression, including myeloid-derived suppressor cells (MDSCs), regulatory T (Treg) cells, cancer-associated fibroblasts (CAFs), and T helper type 2 (Th2) cells (**Figure 10A**) (39). Consistently, the anticancer cells were significantly lower in the high-risk group, such as CD8+ T cells, cytotoxic cells, and effector memory T (Tem) cells (**Figure 10B**). The TIDE method was applied to estimate the ICB resistance score, and the results showed that the low-risk group was more sensitive to ICB treatment (**Figure 10C**).

Notably, the high-risk group patients had higher TMB levels and higher expression levels of immune checkpoints (PDCD1 and PD-L1), which was contradictory to the TIDE estimation (**Figures 8E**, **10C, D**). Then, the “easier” R package was employed to objectively assess the ICB response by leveraging multiple proxies of the immune response, including cancer type, TMB, pathway activities, cell fractions, transfer factor activities, and intracellular and intercellular communications. Interestingly, TMB exhibited a negative correlation with the EaSIeR score (**Figure 10E**). However, there was no significant difference regarding the EaSIeR scores between the high- and low-risk groups (**Figure 10F**).

### 3.7 Loss-of-function experiments in PC cells

**Figure 11:**
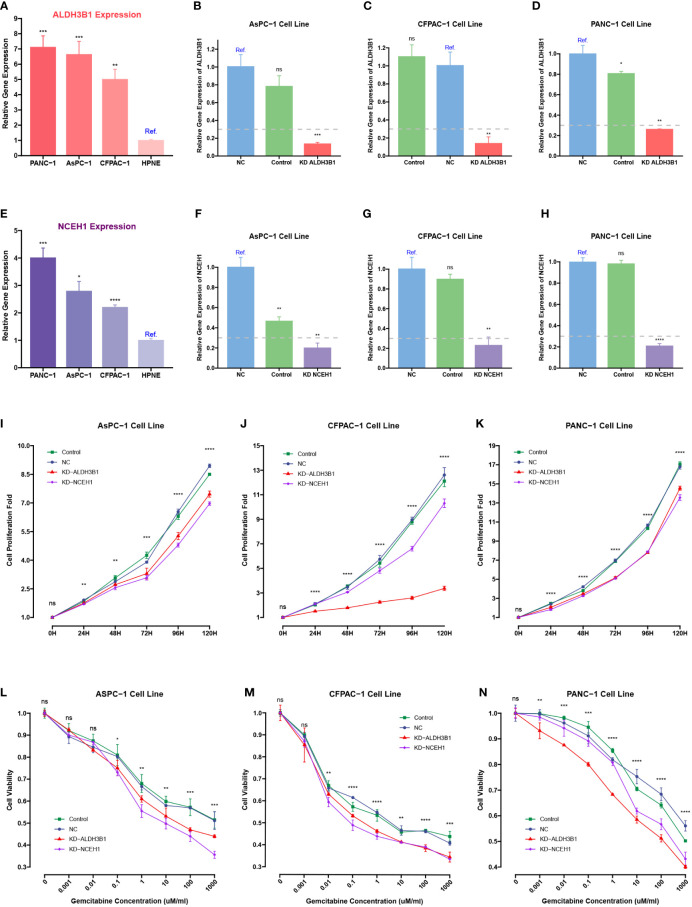
CCK-8 and qRT-PCR assays of pancreatic cancer cells transfected with ALDHB1 and NCEH1 siRNAs. **(A, B)** Relative mRNA expression levels of ALDH3B1 and NCEH1 in three pancreatic cancer cell lines and hTERT-HPNE cells. **(C-H)** Knockdown efficacies of ALDHB1 and NCEH1 siRNAs in AsPC-1 **(C, D)**, CFPAC-1 **(E, F)**, and PANC-1 **(G, H)** cells. **(I-N)** Results of CCK-8 proliferation and cytotoxicity experiments in AsPC-1 **(I, L)**, CFPAC-1 **(J, M)**, and PANC-1 **(K, N)** cells. *, p < 0.05; **, p < 0.01; ***, p < 0.001; ****, p < 0.0001; ns, not statistically significant.

Because previous studies reported that EGFR, ERAP2, and MSLN were associated with gemcitabine response, siRNA-mediated knockdowns were carried out to investigate the roles of NCEH1 and ALDH3B1 in cell proliferation and gemcitabine resistance (40–42). First, RT-qPCR analyses demonstrated that the expression levels of ALDH3B1 and NCEH1 were relatively higher in the three PC cell lines (AsPC-1, CFPAC-1, and PANC-1) than in the normal pancreatic epithelial cell line (hTERT-HPNE) (**Figures 11A, B**). At the mRNA protein level, high silencing efficiencies (> 70% for PCR assay, > 40% for WB assay) of both siRNAs were achieved at 48 hours posttransfection (**Figures 11C-H**; **Supplementary Figure S3A-C**).

The CCK-8 proliferation assay indicated that the proliferation rates of the three PC cell lines were noticeably diminished after knocking down ALDH3B1 and NCEH1 (**Figures 11I-K**, **Supplementary Figure S3D-F**). Gemcitabine cytotoxicity assays demonstrated that knockdown of ALDH3B1 and NCEH1 enhanced gemcitabine sensitivity (**Figures 11L-N**). Of note, this effect was concentration-dependent. The concentration threshold for viability differences was 0.01/0.1/10 for CFPAC-1/AsPC-1/PANC1 cells. These findings indicated that NCEH1 and ALDH3B1 were pivotal regulators of cell proliferation and gemcitabine sensitivity in PC.

## 4 Discussion

PC is an extremely aggressive and lethal malignancy threatening global health. Multiagent cytotoxic regimens are essential for the multidisciplinary management of PC, either alone or in combination with ICBs, surgical resection, and radiation therapy (43). Gemcitabine has been widely used as the first-line agent for progressive and metastatic PC, although it only shows a slight improvement in prognosis (44). The limited therapeutic efficacy of gemcitabine mainly results from intrinsic and acquired chemoresistance. Factors leading to chemoresistance are multifaceted in PC, such as autophagy, epithelial-mesenchymal transition, and upregulation of ATP-binding cassette transport protein (45–47). Therefore, pursuing a comprehensive understanding of chemoresistance mechanisms is conducive to yielding satisfactory therapeutic effects.

In this study, a LASSO Cox risk model and a prognostic nomogram were developed based on the five DEGs upregulated in gemcitabine-resistant PC cells as opposed to gemcitabine-sensitive PC cells, including EGFR, MSLN, ERAP2, ALDH3B1, and NCEH1 (also known as KIAA1363 or AADACL1). This gene signature was an independent prognostic biomarker for predicting prognosis and gemcitabine sensitivity. Except for ALDH3B1 and NCEH1, the other three genes have been reported to be associated with tumor progression and gemcitabine resistance in PC. EGFR inhibitors have been widely studied for their potential applications in PDAC since 2007. Compared with monotherapy, gemcitabine in combination with erlotinib can improve survival in metastatic PC patients, while gemcitabine plus afatinib can impede the growth and metastasis potential of PC stem cells (40, 48). A novel conjugate targeting EGFR/HER2 can enhance gemcitabine sensitivity *via* the SMAD4-mediated mechanism (49). Although MSLN expression is restricted to mesothelial cells in healthy tissues, it is also a malignant factor expressed by 80-85% of PC masses (50). Amatuximab treatment, an MSLN-blocking antibody, can result in a reduction in metastatic potential and apoptosis induced by gemcitabine (51). Additionally, MSLN is also a valuable predictor of responses to EGFR inhibitors and gemcitabine (41, 52). Knockdown of ERAP2 can enhance the cytotoxicity of gemcitabine against PC cells and compromise the capacity of migration and invasion (42). Moreover, ERAP2 can indirectly modulate PC aggressiveness. Pancreatic stellate cells (PSCs) are the most predominant contributors to the hyperdense stroma that favors tumor progression (53). Decreased ERAP2 expression weakens the activation of PSCs, tumor-PSC interactions, and the capacities of PSCs to promote cancer aggression (54). ALDH3B1 is a critical enzyme against oxidative stress (55). After silencing ALDH3B1, glioma cells suffer from G2/M arrest and epithelial-mesenchymal transition (EMT) inhibition (56). NCEH1 is a transmembrane hydrolase involved in neutral ether lipid metabolism (57). It has been reported that the inactivation of NCEH1 impairs cell migration and tumor growth in ovarian and prostate cancers (57, 58). Although no experiments have investigated the roles of ALDH3B1 and NCEH1 in PC, our bioinformatic and experimental analyses indicated that the increased expression of both genes was positively correlated with PC progression and gemcitabine chemoresistance, thus conferring a survival disadvantage. Taken together, all five genes comprising this established signature are experimentally or clinically confirmed as indicators of PC progression and gemcitabine sensitivity.

In addition to gemcitabine, this gene signature was theoretically associated with sensitivities to other first-line anti-PC agents, including doxorubicin, olaparib, paclitaxel, and FOLFRINOX. The mechanisms of multidrug chemoresistance in the high-risk group might be accounted for by the high activities of pathways associated with mismatch repair (59), cell cycle (60), spliceosome (61), hypoxia (62), and multiple oncogenic signaling (P53, MYC, TGF-beta, and PI3K/Akt) (63–66).

The accumulation of multiple somatic mutations drives the cancerous transformation of normal pancreatic duct cells, especially four frequently mutated genes (KRAS, CDKN2A, TP53, and SMAD4) (67). Consistently, our results suggested that the high-risk patients harbored higher TMB levels and higher mutation frequencies of these four genes than their low-risk counterparts. TMB is conventionally used as a biomarker to predict ICB response across various cancer types, but its relevance to PC chemosensitivity remains largely unknown (68). In the JIPANG study, nonsquamous non-small cell lung cancer (Ns-NSCLC) patients with high TMB levels (≥ 12 mut/Mb) tended to benefit more from pemetrexed plus cisplatin treatment than from vinorelbine plus cisplatin (69). One retrospective analysis revealed that TMB-low patients with colorectal cancer were more sensitive to irinotecan-based chemotherapy versus oxaliplatin-based chemotherapy (70). However, another study pointed out that TMB was not significantly correlated with clinical benefits in breast, lung, and gastrointestinal cancers (71). Therefore, the predictive performance of TMB for drug sensitivity depends on the tumor type. In our study, TMB was positively correlated with multichemoresistance in PC, which might result from the gradual acquisition of resistance mutations. For example, KRAS mutations (72) can impair the efficacy of anti-EGFR treatment in colorectal cancer, and gemcitabine resistance is linked to TP53 mutations (73), CDKN2A inactivation (74), and SMAD4 loss (75). Thus, TMB is a molecular feature to predict the chemotherapy response of PC.

Advances in immunotherapies have radically changed the therapy options for some historically chemotherapy-refractory malignancies. However, PC has several intrinsic properties resulting in immune escape. Our TME analyses indicated that high-risk PC patients were more immune-cold with less CD8+ T cell infiltration and more protumor cell infiltration (MDSCs, CAFs, Treg cells, and Th2 cells), which was confirmed by the TIDE estimation. Typically, immune-cold tumors are more resistant to ICB treatment than immune-hot tumors (76). However, we observed that the high-risk group had higher TMB levels and expression levels of immune checkpoints (PDCD1 and PD-L1). TMB is a proxy of tumor antigenicity, and high-TMB patients tend to respond favorably to immunotherapy (68). TMB, in concert with CD274 (PD-L1) expression, serves as a biomarker panel for ICB selection in many kinds of cancers (77). Then, we used the “easier” package to predict the ICB response, taking into account intrinsic and extrinsic immune escape mechanisms. Paradoxically, the risk score was negatively correlated with TMB. This may be caused by KRAS mutation, which is present in 81-92% of PC patients (78, 79). Mutated KRAS contributes to the development of the immunosuppressive TME in PC through several avenues, including recruitment of MDSCs and Treg cells (80, 81), maintenance of the fibroinflammatory stroma (82), induction of Th17 cells (83), and upregulation of PD-L1 expression *via* mRNA stabilization (84). The EaSIeR scores (ICB efficacies) of high-risk patients were similar to those of low-risk patients. Despite the successes of ICB in other tumors, the overall response rates of PC patients receiving anti-PD-L1 monotherapy and anti-CTLA-4 plus anti-PD-L1 regimen are reported to be 0% and 3.1%, respectively. Thus, all the high- and low-risk patients were not responsive to ICB treatment alone. Combination strategies are more likely to yield satisfactory therapeutic effects, such as pegvorhyaluronidase alfa with nab-paclitaxel plus gemcitabine (85).

Although we performed comprehensive analyses and experimental validations, there are several limitations that warrant caution. Firstly, since the RNA-seq and chemosensitivity data were acquired from different sources, some important genes might have been ignored and not incorporated into this gene signature. Secondly, we only validated ALDH3B1 and NCEH1 mediated gemcitabine resistance in PC cell lines. Further studies are needed to investigate the roles of ALDH3B1 and NCEH1 in chemoresistance to doxorubicin, olaparib, paclitaxel, fluorouracil, irinotecan, and oxaliplatin. Additional research is essential to investigate the specific roles of ALDH3B1 and NCEH1 in these chemotherapeutics. Thirdly, only bioinformatic software was employed to predict responses to chemotherapy and immunotherapy. Further clinical studies are warranted to validate the predictive performance of this chemoresistance-related signature.

In summary, this study identified a chemoresistance-related gene signature that can independently facilitate prognosis prediction in PC. This gene signature also helps to distinguish immune features and is correlated with TMB. Additionally, ALDH3B1 and NCEH1 are two promising targets for treating PC.

## Data availability statement

The original contributions presented in the study are included in the article/**Supplementary Material**. Further inquiries can be directed to the corresponding author.

## Author contributions

XT and JC conceived and planned this study. ZL, JC, ZW, and WL helped interpret the results. JC took the lead in writing the manuscript. All authors contributed to the article and approved the submitted version.
