# Supplementary material for: Identification of a chemoresistance-related prognostic gene signature by comprehensive analysis and experimental validation in pancreatic cancer
Source: Front Oncol. 2023 May 12;13:1132424. doi: 10.3389/fonc.2023.1132424 (PMC10213255; doi:10.3389/fonc.2023.1132424)
Supplement: Supplementary file 1 [file DataSheet_1.zip › Supplementary Material/Supplementary Figure Legends.docx]

# Supplementary Figure Legends

# Supplementary Figure S1: Workflow diagram of current analyses.

# Supplementary Figure S2: Bar plot depicting the gemcitabine resistance scores of 30 PC cell lines.

# Supplementary Figure S3: CCK-8 and Western blot assays of pancreatic cancer cells transfected with siRNAs.

**(A-C)** Western blot assay validating the knockdown efficacies of ALDHB1 and NCEH1 siRNAs in AsPC-1, CFPAC-1, and PANC-1 cells. **(D-F)** Bar plots showing the results of CCK-8 cytotoxicity experiments in AsPC-1 **(D)**, CFPAC-1 **(E)**, and PANC-1 **(F)** cells. *, p < 0.05; **, p < 0.01; ***, p < 0.001; ns, not statistically significant.
