# Supplementary figures and images for: Identification of a chemoresistance-related prognostic gene signature by comprehensive analysis and experimental validation in pancreatic cancer
Source: Front Oncol. 2023 May 12;13:1132424. doi: 10.3389/fonc.2023.1132424 (PMC10213255; doi:10.3389/fonc.2023.1132424)

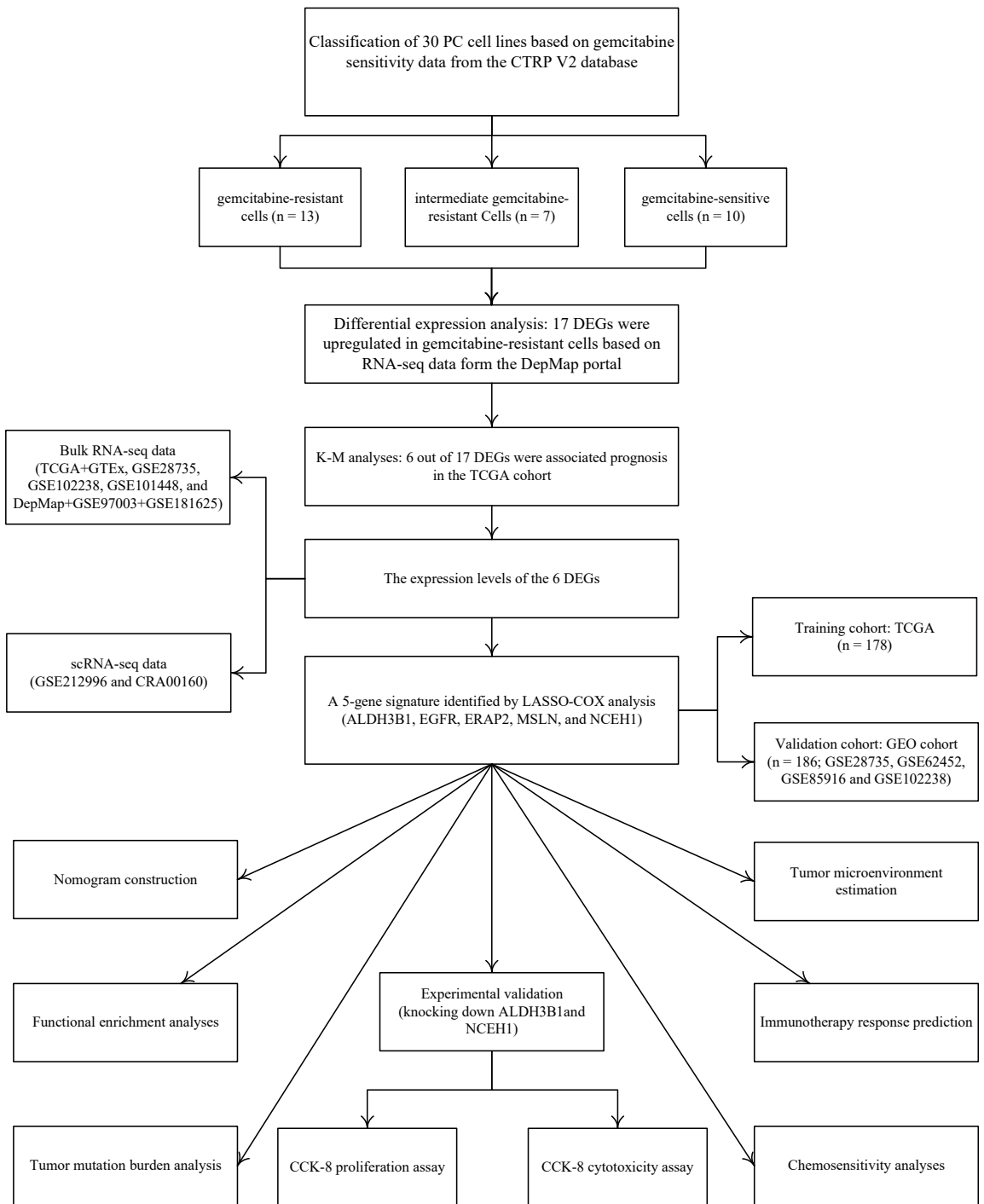

Supplement: Supplementary file 1 [file DataSheet_1.zip › Supplementary Material/Supplementary Figure S1.pdf]

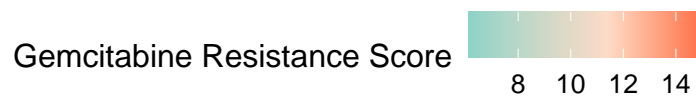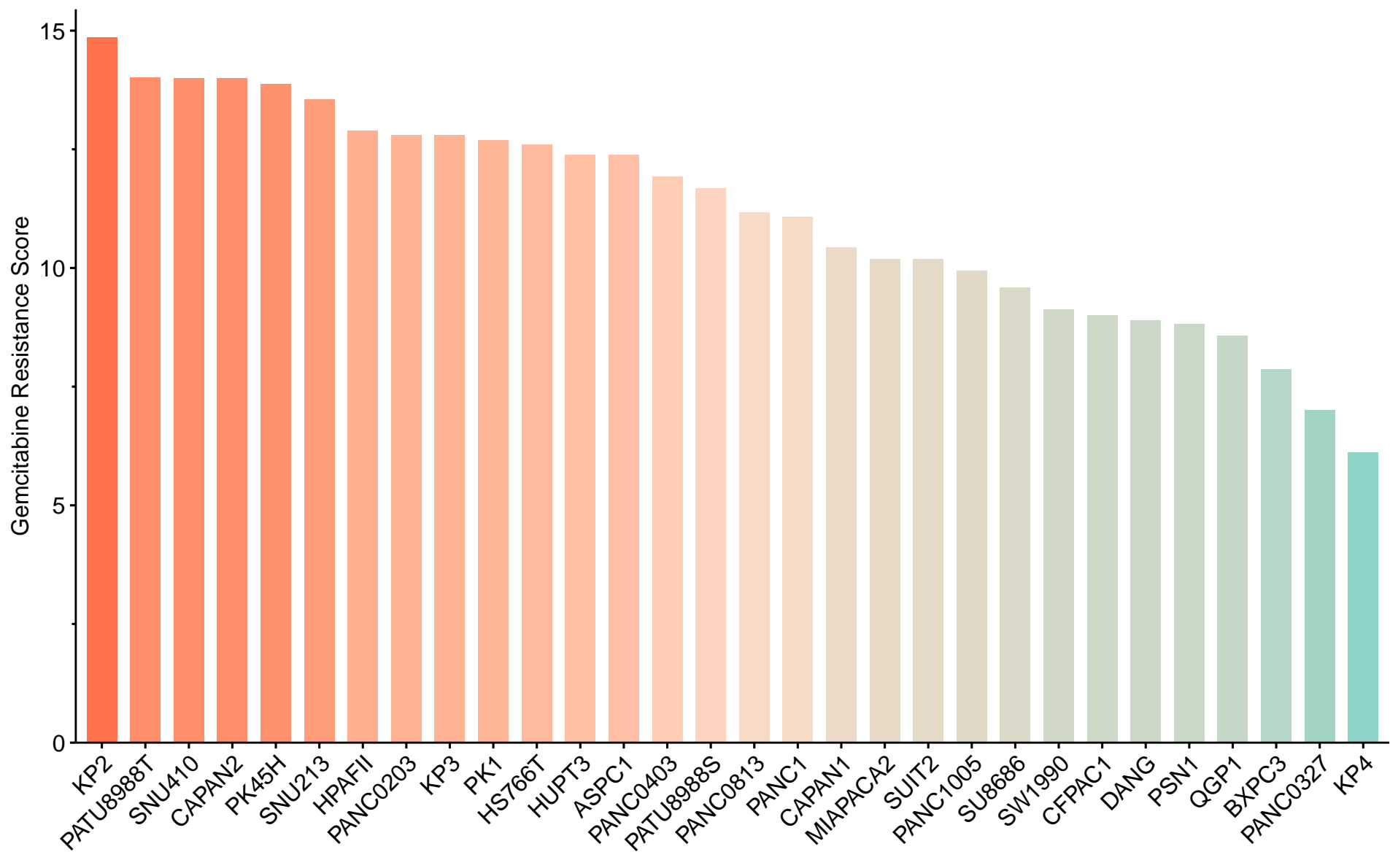

Supplement: Supplementary file 1 [file DataSheet_1.zip › Supplementary Material/Supplementary Figure S2.pdf]

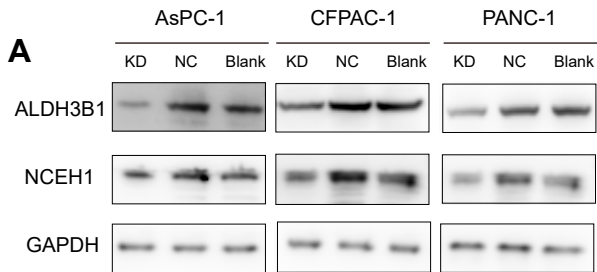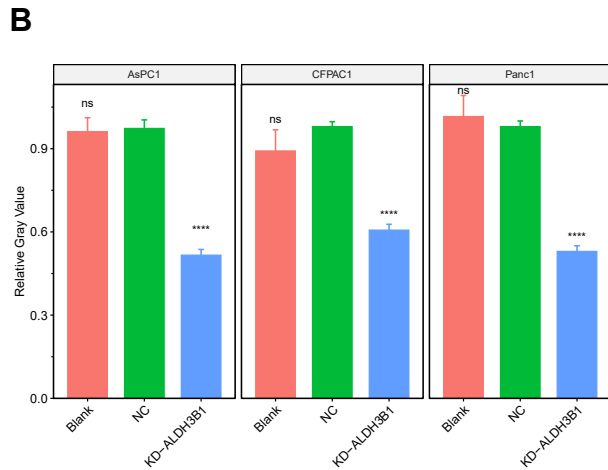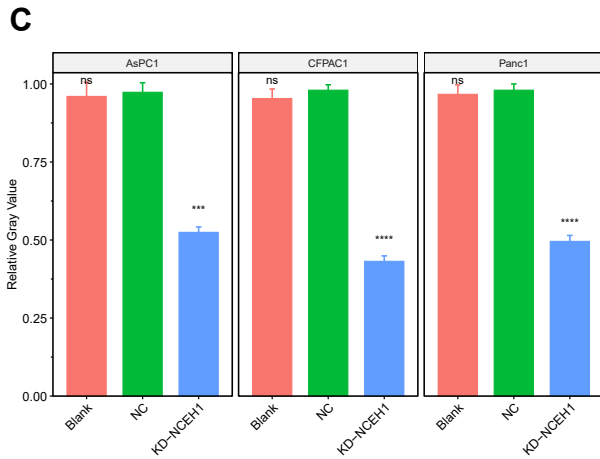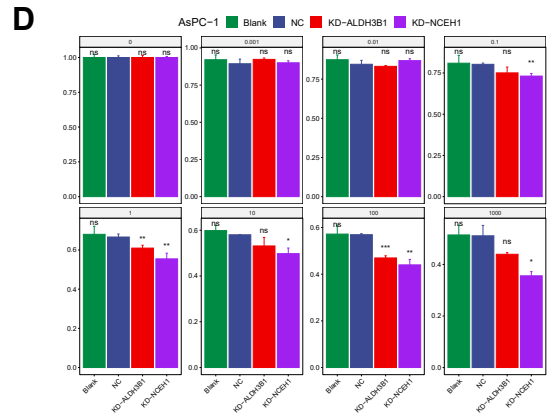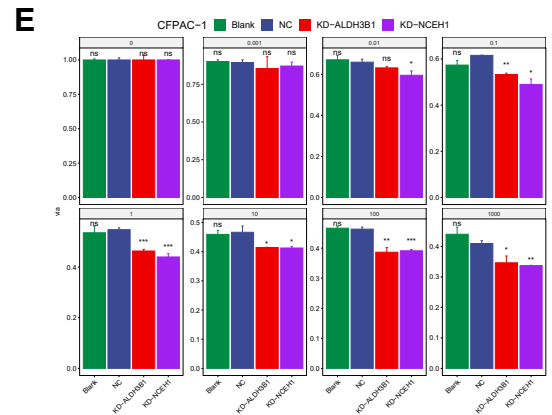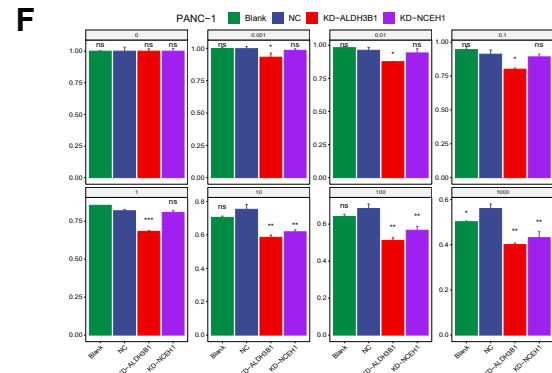

Supplement: Supplementary file 1 [file DataSheet_1.zip › Supplementary Material/Supplementary Figure S3.pdf]
